# Carbon nanoparticles induce endoplasmic reticulum stress around blood vessels with accumulation of misfolded proteins in the developing brain of offspring

**DOI:** 10.1038/s41598-020-66744-w

**Published:** 2020-06-22

**Authors:** Atsuto Onoda, Takayasu Kawasaki, Koichi Tsukiyama, Ken Takeda, Masakazu Umezawa

**Affiliations:** 1 Faculty of Pharmaceutical Sciences, Sanyo-Onoda City University, 1-1-1 University Street, Sanyo-Onoda city, Yamaguchi 756-0884 Japan; 2 Department of Pediatrics, Nagoya University Graduate School of Medicine, 65 Tsurumai-cho, Showa-ku, Nagoya city, Aichi 466-8560 Japan; 3 Research Fellow of Japan Society for the Promotion of Science, 5-3-1 Kouji-machi, Chiyoda-ku, Tokyo 102-0083 Japan; 4 Infrared Free Electron Laser Research Center, Research Institute for Science and Technology, Organization for Research Advancement, Tokyo University of Science, 2641 Yamazaki, Noda, Chiba 278-8510 Japan; 5 Department of Chemistry, Faculty of Science, Tokyo University of Science, 1-3 Kagurazaka, Shinjuku-ku, Tokyo 162-8601 Japan; 6 Department of Materials Science and Technology, Faculty of Industrial Science and Technology, Tokyo University of Science, 6-3-1 Niijuku, Katsushika-ku, Tokyo 125-8585 Japan

**Keywords:** Risk factors, Occupational health, Neurological disorders

## Abstract

Nano-particulate air pollution threatens developing brains and is epidemiologically related to neurodegenerative diseases involving deposition of misfolded proteins. However, the mechanism underlying developmental neurotoxicity by nanoparticles remains unknown. Here, we report that maternal exposure to low doses of carbon black nanoparticle (CB-NP) induces endoplasmic reticulum (ER) stress associated with accumulation of misfolded proteins. Notably, offspring specifically showed high induction of ER stress in perivascular macrophages and reactive astrocytes only around brain blood vessels, along with accumulation of β-sheet-rich proteins regarded as misfolded proteins. Our results suggest that maternal CB-NP exposure induced ER stress in PVMs and reactive astrocytes around blood vessels in the brain of offspring in mice. The induction of ER stress accompanied by the perivascular accumulation of misfolded proteins is likely to be associated with perivascular abnormalities and neurodegeneration, and development of neurodegenerative diseases related to particulate air pollution.

## Introduction

The health impacts of air pollution are widely recognized as a global problem that requires immediate solution^1^. Adverse health effects of particles in the atmosphere, such as fine particulate matter (PM2.5) and ultrafine particles (nanoparticles), have received much attention in toxicological sciences^2^. In particular, developmental neurotoxicity of nanoparticles is a major issue in toxicology^3,4^. Epidemiological evidence suggests that exposure to particulate air pollution including nanoparticles is likely to increase risk for onset of neurodegenerative diseases, such as Alzheimer’s disease and Parkinson’s diseases, later in life of offspring^5–7^. Proteopathies, such as neurodegenerative diseases, are a class of conformational diseases that generally display deposition of misfolded proteins consisting of multiple β-sheet structures. Nanoparticles derived from air pollution have been detected from the brains of patients with Alzheimer’s disease^8^. Moreover, amounts of nanoparticles in the brain correlate with the incidence of Alzheimer’s disease^9^. Also, animal and epidemiological studies have revealed that maternal exposure to nanoparticles disturbs brain development and induces abnormal cognitive functions in the offspring^10,11^. However, the mechanisms underlying developmental neurotoxicity due to low doses of nanoparticles remain largely unknown. It is critical to elucidate these mechanisms in order to establish prevention strategies and medical treatments for brain disorders.

To reveal key events underlying the mechanisms of the developmental neurotoxicity, we previously exposed pregnant mice to low doses of carbon black nanoparticle (CB-NP), a model particle of air pollution^12^, through the respiratory tract and investigated the effects of CB-NP exposure on the brains of offspring^13–16^. Maternal CB-NP exposure diffusely injured brain perivascular macrophages (PVMs), which play a role in clearance of waste including misfolded proteins^17^, and adjacent astrocytes, which play a role in maintenance of cerebrovascular homeostasis^18^. In particular, over-activation of astrocytes (astrogliosis) and decreases in normal PVMs in the brain were sensitively observed in various brain regions following maternal exposure to low doses of CB-NP^13–15^. These results indicate that perivascular tissues are one of the most vulnerable to maternal CB-NP exposure. Importantly, initial abnormalities of brain perivascular areas contribute to the development of neurodegenerative diseases^19^. Thus, causes of the perivascular abnormalities may be key events underlying nanoparticle-induced developmental neurotoxicity. As one of the causes, deposition of misfolded proteins in brain perivascular areas, reminiscent of proteopathies including neurodegenerative diseases, is suggested by our previous findings captured by *in situ* Fourier transform infrared spectroscopy (*in situ* FT-IR)^16^. We hypothesized that if deposition of misfolded proteins is induced by maternal CB-NP exposure, endoplasmic reticulum (ER) stress caused by accumulation of misfolded proteins is likely to occur in the brain. Therefore, the purpose of the current study is to investigate the ER stress along with accumulation of misfolded proteins and relationship between the ER stress and perivascular abnormalities in the brain of offspring mice maternally exposed to CB-NP.

## Methods

### Preparation of nanoparticle suspension

Insoluble CB-NP Printex 90 was purchased from Degussa Ltd (Frankfurt, Germany). CB-NP had an average primary particle size of 14 nm with a specific surface area of 295–338 m^2^/g. Purity of CB-NP was >99% (carbon: >99 wt%, nitrogen: 0.82 wt%, hydrogen: 0.01 wt%, and organic impurity contents: <1%wt). CB-NP was suspended at 5 mg/mL in ultra-pure water, sonicated for 30 min, and immediately filtered through a 450-nm filter (S-2504; Kurabo Co. Ltd., Osaka, Japan) before intranasal instillation, as previously described^13^. Hydrodynamic diameter of CB-NP in suspension was determined by dynamic light scattering (NANO-ZS, Sysmex Co., Kobe, Hyogo, Japan) with Rayleigh-Debye equation and transmission electron microscopy (JEM 1200EXII, JEOL Ltd., Akishima, Tokyo, Japan) on collodion-coated 200 Cu mesh (No. 6511, Nisshin EM, Shinjuku, Tokyo, Japan). Filtered CB-NP in suspension showed small agglomerated particles with a peak size of 84.2 nm and poly-dispersity index of 0.143^13^. This size corresponds well with the typical small agglomerate sizes observed in CB-NP samples^13^. The size distribution of the CB-NP suspension was not altered until use for the intranasal instillation. CB-NP concentration in the suspension was 95 μg/mL by peak area of carbon signal (2.77 keV) obtained using a field emission scanning electron microscope (JSM-6500F) with an attached energy-dispersive X-ray analyzer (JSM-6500F)^13^.

### Animals and treatments

Twenty pregnant ICR mice at 11 weeks of age (gestational day 3) were purchased from SLC Inc. (Hamamatsu, Shizuoka, Japan). The mice were randomly assigned to CB-NP (n = 10) and control (n = 10) groups. The mice in each group were housed in cages (1 pregnant mouse/cage) under controlled temperature (22–24 °C) and humidity (50–60%) with a 12-h dark/light cycle and ad libitum access to food and water. Pregnant mice were treated with intranasal instillation of CB-NP suspension at 95 μg/mL/kg body weight (CB-NP group) or with distilled water (control group) under anesthesia with isoflurane on gestational day 5 and 9. After birth, the number of pups per dam was adjusted to 10 on postnatal day 1. The pups were housed in cages (3 pups/cage) under the same conditions after weaning for 3 weeks. We randomly chose one male offspring per one dam and collected the brain from the selected offspring at 6 weeks after birth (n = 10 in each group). Only male offspring were used to reduce effects of sexual cycle. All experiments were performed in accordance with Animal Research: Reporting *In Vivo* Experiments guidelines for the care and use of laboratory animals^20^ and were approved by Tokyo University of Science’s Institutional Animal Care and Use Committee (Approval Number: Y16057). All sample collection was performed under isoflurane anesthesia, and all efforts were made to minimize the number of mice used and their suffering.

### Preparation of serial sections of mouse brains

Six-week-old mice anesthetized by isoflurane were transcardially perfusion with phosphate buffered saline (PBS) followed by perfusion-fixation with 4% paraformaldehyde in 0.1 M phosphate buffer. Brains were collected after perfusion-fixation, post-fixed in 4% paraformaldehyde in 0.1 M phosphate buffer for 20 h, and subsequently cryoprotected in phosphate-buffered sucrose solutions (10% sucrose, 4–6 h; 20% sucrose, 4–6 h; 30% sucrose, 12–24 h) containing 0.1% sodium azide. Sections were then embedded in Tissue-Tek OCT compound (Sakura Finetek Japan Co., Ltd, Tokyo, Japan) and immediately frozen in Histo-Tek Hyfluid (Sakura Finetek Japan Co., Ltd) at −80 °C. Serial sections (10-μm thick) were prepared from the frozen blocks using a Tissue-Tek Polar instrument (Sakura Finetek Japan Co., Ltd), and mounted onto an IR-reflective stainless-steel base for *in situ* FT-IR or a glass slide for immunofluorescence. Sections were air-dried for 30 h before measurement and staining to prevent interference due to moisture.

### *In situ* FT-IR

The detail method was previously described^16^. Briefly, for *in situ* FT-IR measurements, we used an IRT-7000 IR microscope combined with an FT/IR-6100 spectrometer (Jasco Co., Tokyo, Japan). Spectra were acquired in reflection mode using a 16× Cassegrain lens and collected in the mid-IR range of 700–4000 cm^−1^ at a resolution of 4 cm^−1^ over 64 scans from 30 × 30-μm apertures. The reflection spectra were obtained from tissues around brain blood vessels in the cerebral cortex using lattice measurement (x-axis: 7 points, y-axis: 7 points, total of 49 spectra acquired). Sixty-four spectra were acquired from only embedding medium (OCT compound) regions and an average of these spectra was used as a common basal line for analysis of FT-IR spectral data. Smoothing and normalization of the obtained spectra were performed on the region containing the amide bands (1000–2000 cm^−1^) using Spectra Manager Software Ver. 2 (Jasco International Co., Ltd, Tokyo, Japan). We deconvoluted the spectra for protein secondary structural analysis and calculated ratios of secondary structure contents (α-helix, β-sheet, β-turn, and random coil) from peak intensities of the amide I bands (1600–1700 cm^−1^). The calculated ratios of secondary structures were visualized using the universal RGB code on the protein mapping analysis software (IR-SSE; JASCO Co., Ltd.)^21^.

### Immunofluorescence

Serial sections were stained using immunofluorescent antibodies following standard methodology to evaluate protein expression of glial fibrillary acidic protein (GFAP) as an astrocyte activation marker, macrophage mannose receptor (MMR/CD206) as a selective marker of PVMs, activating transcription factor 6 (ATF6) and C/EBP-homologous protein (CHOP/GADD153/DDIT3) as endoplasmic reticulum stress markers^22,23^. Sections were submerged in PBS to remove the Tissue-Tek OCT compound and then blocked by 10% normal donkey serum (IHR-8135, Immunobioscience, Mukilteo, WA, USA) for 1 h at room temperature. Sections were then incubated with primary goat polyclonal anti-GFAP antibody (1:500 in PBS; code no. ab53554, Abcam, Cambridge, UK) or goat polyclonal anti-MMR antibody (1:200 in PBS; code no. AF2535, R&D Systems, Minneapolis, MN, USA) for 16 h at 4 °C. After rinsing 3 times for 5 min per a rinse with PBS, sections were incubated with secondary Dylight 488-conjugated donkey anti-goat IgG (1:1000 in PBS; code-no. 605-741-125, Rockland Immunochemicals Inc., PA, USA) for 120 min at room temperature. After rinsing 3 times for 5 min per a rinse with PBS, sections were incubated with primary rabbit polyclonal anti-ATF6 antibody (1:100 in PBS; code no. ab37149, Abcam) or rabbit polyclonal anti-CHOP antibody (1:100 in PBS; code no. PAB8734, Abnova, Taipei, Taiwan) for 16 h at 4 °C. After rinsing 3 times for 5 min per a rinse with PBS, sections were further incubated with secondary Dylight 649-conjugated donkey anti-rabbit IgG (1:1000 in PBS; code-no. 611-743-127, Rockland Immunochemicals Inc.) for 120 min at room temperature. Sections were then rinsed three times for 5 min per rinse with PBS and twice for 5 min per rinse with distilled water. Nuclei were counterstained using Hoechst 33342 (code no. 346–07951, Dojindo Laboratories, Kumamoto, Japan).

### Cell counting

The expression of GFAP, MMR, ATF6, and CHOP was evaluated using a fluorescent microscope (BZ-9000, Keyence Co., Osaka, Japan) to detect and quantify ATF6-positive astrocytes, CHOP-positive astrocytes, ATF6-positive PVMs, and CHOP-positive PVMs in the cerebral cortex. For each brain obtained from offspring mice, one hundred sections (total 1,000 μm) were prepared from bregma of cerebrum to olfactory bulb along the coronal plane. One of every five sections (twenty sections per mouse; about 20 mm^2^/mouse at 50-μm intervals) was chosen for the quantitative analysis of cells positive for GFAP, MMR, ATF6, and CHOP. Cell counting was done under fluorescence microscopy (BZ-9000, Keyence Co., Osaka, Japan) at 200× magnification and calculated per mm^2^ area. All scale bars in pictures represent 40 μm.

### Statistical analysis

All data are expressed as means in the form of box-and-whisker diagrams. The numbers of ATF6-positive astrocytes, ATF6-positive PVMs, and CHOP-positive PVMs were analyzed using Mann-Whitney’s U test. Proportions of secondary structure contents were evaluated using Kruskal-Wallis test followed by Steel-Dwass post hoc test. The level of significance was set at p < 0.05. Statistical analyses were carried out using R version 3.5.3 (https://www.r-project.org/).

### Ethics approval and consent to participate

All experiments were performed in accordance with Animal Research: Reporting *In Vivo* Experiments guidelines for the care and use of laboratory animals20 and were approved by Tokyo University of Science’s Institutional Animal Care and Use Committee (Approval Number: Y16057). All sample collection was performed under isoflurane anesthesia, and all efforts were made to minimize the number of mice used and their suffering.

## Results

**Figure 1:**
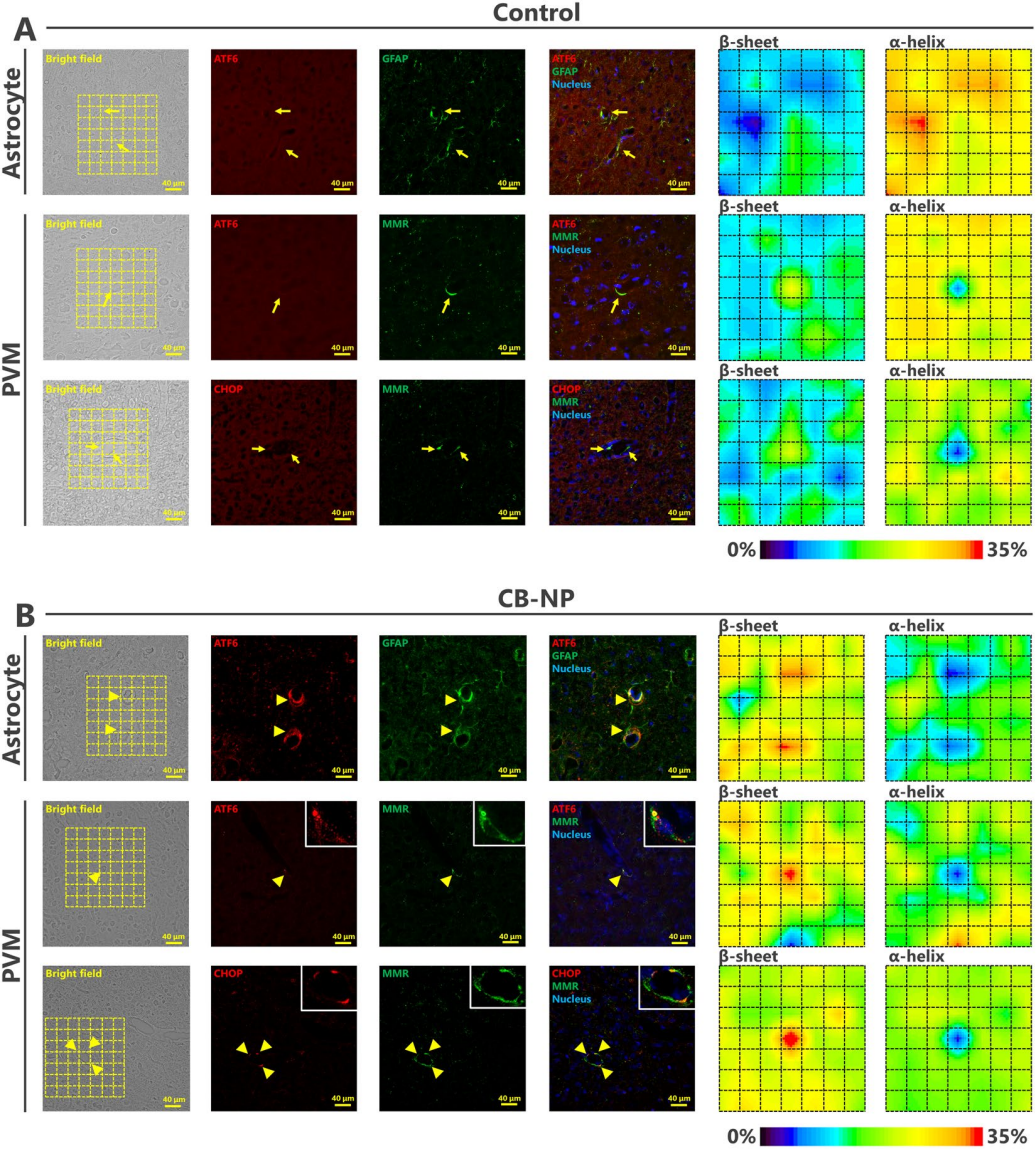
Histopathology of ER stress in the brain of mice maternally exposed to CB-NP and mapping analysis of protein secondary structures. (**A,B**) Representative fluorescent micrographs of astrocytes (top) and PVMs (middle and bottom) and mapping images obtained from FT-IR spectra of the lattices, shown on left figures, on frozen brain sections. (**A**) Control group. Arrows indicate astrocytes weakly expressing GFAP and PVMs expressing MMR. (**B**) CB-NP group. Arrowheads indicate astrocytes strongly expressing GFAP with ATF6 and MMR-positive PVMs expressing ATF6 or CHOP around blood vessels. All scale bars are 40 μm. [Abbreviations] ATF6: activating transcription factor 6, CB-NP: carbon black nanoparticle, CHOP: C/EBP-homologous protein, ER: endoplasmic reticulum, FT-IR: Fourier transform infrared spectroscopy, GFAP: glial fibrillary acidic protein, MMR: macrophage mannose receptor, PVM: perivascular macrophage.

**Figure 2:**
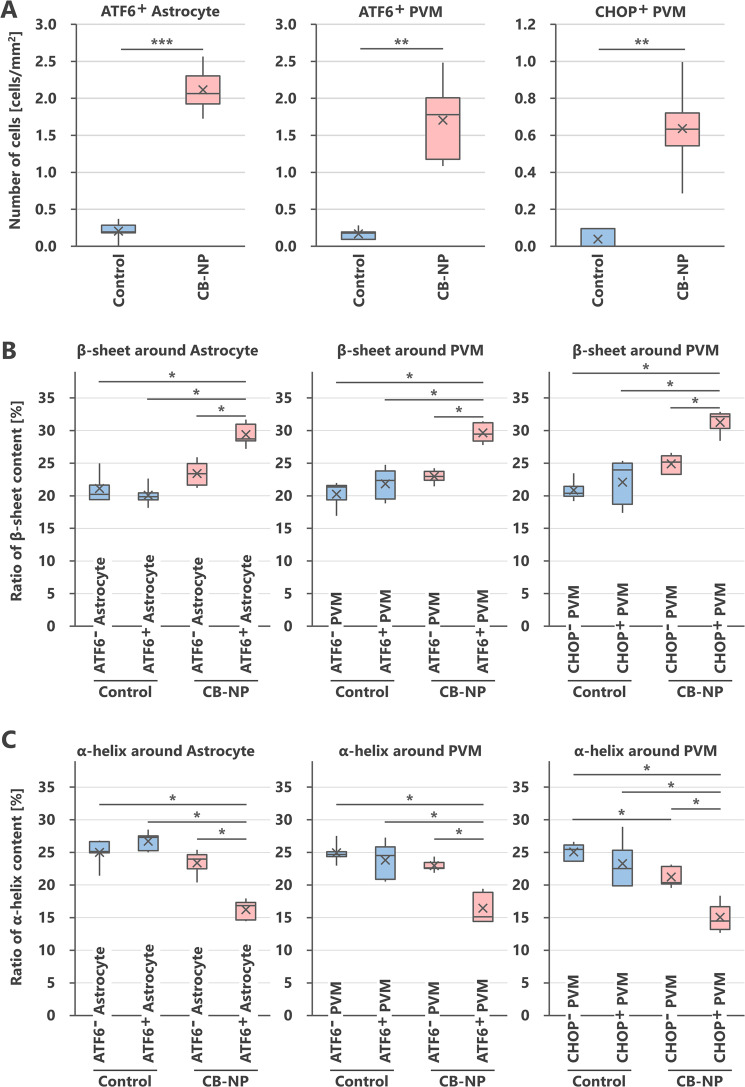
Number of astrocytes and PVMs expressing ER stress markers in the brain of mice maternally exposed to CB-NP and ratios of protein secondary structures around the cells. (**A**) Numbers of astrocytes (GFAP-positive) and PVMs (MMR-positive) highly expressing ATF6 and CHOP in the brain. (**B**,**C**) β-sheet and α-helix contents in areas close to blood vessels containing ATF6-negative and -positive astrocytes, ATF6-negative and -positive PVMs, and CHOP-negative and -positive PVMs in the control and CB-NP group, respectively. Data are presented as box-and-whisker plots with mean (×) of biological quadruplicates. *p < 0.05, **p < 0.01, and ***p < 0.001. [Abbreviations] ATF6: activating transcription factor 6, CB-NP: carbon black nanoparticle, CHOP: C/EBP-homologous protein, ER: endoplasmic reticulum, GFAP: glial fibrillary acidic protein, MMR: macrophage mannose receptor, PVM: perivascular macrophage.

We investigated expression levels of ATF6 and CHOP as ER stress markers of astrocytes and macrophages^22,23^, and found that maternal CB-NP exposure specifically upregulate the ER stress markers around brain blood vessels, but not within parenchyma far from blood vessels (Fig. 1). The expression level of GFAP increased in the CB-NP group compared to the control group as reported in previous studies^10,13–15^. Thus, to evaluate the expression of the ER stress markers in the reactive astrocytes highly expressing GFAP, the marker proteins were visualized by double-immunofluorescence with GFAP. High ATF6 expression was particularly observed in the reactive astrocytes around brain blood vessels in the exposure group. In contrast, no expression of ATF6 was observed in the astrocytes of the control group, even in the case of GFAP-positive astrocytes. The ATF6 upregulation was also observed in PVMs (macrophage-mannose-receptor positive cells). Furthermore, PVMs also expressed CHOP in the exposure group. The number of ATF6-positive astrocytes, ATF6-positive PVMs, and CHOP-positive PVMs was significantly increased after maternal exposure to low doses of CB-NP (Fig. 2A). In contrast, high expression of ATF6 and CHOP were not observed in neurons and other glial cells in the brains of control and CB-NP exposure groups.

Next, we performed *in situ* FT-IR analysis combined with immunofluorescence to investigate protein structures on serial sections of the brain. First, brain perivascular tissues where ER stress was induced in the maternal CB-NP exposure group displayed high *in situ* FT-IR signals derived from β-sheet structures (Fig. 1). Second, β-sheet structures were specifically and significantly increased in the areas around blood vessels with ATF6-positive astrocytes, ATF6-positive PVMs, and CHOP-positive PVMs compared with other areas around blood vessels (Fig. 2B). Additionally, α-helix structures were significantly decreased around these blood vessels (Fig. 2C). In the control group, there was no difference between the ratios of these secondary structures around brain blood vessels with and without ER stress (Fig. 2B,C). Thus, this study shows that β-sheet-rich proteins increase and accumulate only around blood vessels with astrocytes and PVMs strongly expressing ER stress markers in the brain of mice maternally exposed to CB-NP.

## Discussion

Here, we report that maternal exposure to 95 μg CB-NP/kg/day induces ER stress related to deposition of misfolded proteins. Notably, offspring specifically showed high expression of ER stress markers (ATF6 and CHOP) in PVMs and reactive astrocytes only around brain blood vessels, along with accumulation of β-sheet-rich proteins. The signals of β-sheet-rich proteins around these brain blood vessels were higher than the ones without reactive astrocytes as previously reported^16^. The concentration of CB-NP used in the current study is relevant for occupational settings because the estimated limit concentration for workers is approximately 480 μg CB-NP/kg/day^14^. In addition, the concentration used in the current study is similar to the one of previous study which have shown little effect on maternal lungs^15^ and placenta^14^. Therefore, the evidence suggests that the low-dose exposure to nanoparticles may have potentially induced the ER stress and brain perivascular abnormalities in the developing brains of children in the real world.

There are many ER stress markers such as IRE1, PEAK, and XBP1^24^. Among these ER stress markers, ATF6 was chosen in the present study because it is suitable for investigating the relationship between ER stress and GFAP overexpression of astrocytes as previously reported^25,26^. Also, ATF6 is one of the principal sensors which sensitively respond to ER stress^24^. While ATF6 is upregulated by mild ER stress, CHOP upregulation is observed upon severe ER stress conditions closely related to cell death of macrophages^27^. Previous findings suggested that the cell death of PVMs is likely to be induced by maternal CB-NP exposure^13^. Therefore, CHOP was also chosen as the ER stress marker that should be examined. Further investigations of more ER stress markers are needed to prospect biological responses and activated pathway after ER stress induction. Because ER stress is closely linked to autophagy and apoptosis^27,28^, they would be important targets of future research on the developmental neurotoxicity of nanoparticles.

ATF6 is generally upregulated by accumulation of misfolded proteins in cells^22^, and is known to activate astrocytes and increase GFAP expression to protect brain tissues^25^. In the current study, the upregulation of ATF6 and GFAP was co-localized in astrocytes, suggesting that ATF6 may promote astrocyte activation to protect the brain from CB-NP-induced injuries. In fact, previous studies demonstrate that maternal CB-NP exposure induces reactive astrogliosis with dose-dependent upregulation of GFAP and end-feet swelling with high expression of water channel (Aquaporin 4)^13,14^. In addition, emerging evidence shows that astrocyte activation is mediated by autophagy following maternal exposure to nanoparticles^26^. Because autophagy is often caused by ER stress^28^, the induction of astrogliosis and ER stress by maternal CB-NP exposure may closely related to the autophagy in astrocytes. Furthermore, CHOP upregulation is observed upon accumulation of misfolded proteins and under conditions of severe ER stress leading to apoptosis^23^. Thus, apoptosis may be induced in CHOP-positive PVMs due to exposure to CB-NP. Consistent with this idea, previous study demonstrates that maternal CB-NP exposure induces histopathological injuries in PVMs adjacent reactive astrocytes and a decrease in the number of PVMs in almost all brain regions^13^. Also, another study indicates positive regulation of angiogenesis-related genes in the brain was induced by maternal CB-NP exposure^14^. The enhanced sign of angiogenesis may be caused by ER stress because ER-stress lead to angiogenesis^29^. Therefore, ER stress is likely to underlie these perivascular abnormalities including astrogliosis with PVM denaturation and may be a key event in the developmental neurotoxicity induced by maternal exposure to nanoparticles. Furthermore, ER stress is generally induced by endogenous misfolded proteins and accumulation of extracellular misfolded proteins associated with neurodegenerative diseases^30–32^. Together, our findings suggest that the β-sheet-rich proteins which accumulate following maternal CB-NP exposure are misfolded proteins, which induce the ER stress in astrocytes and PVMs.

The activation of the ATF6 pathway generally promotes transcription of CHOP^27^. The relationship between ATF6 and CHOP in astrocytes should be evaluated by further studies, but it may be difficult to assess the CHOP expression in astrocytes by histological analysis. We cannot detect the CHOP in the astrocytes by immunofluorescence in the present study. Only one article has displayed the CHOP expression in astrocytes by immunofluorescence, but the expression signal of CHOP in the astrocytes looks very low^33^. Also, the ER stress observed in the astrocytes and PVMs may also have protectively worked for neurons and other brain tissues. Indeed, previous studies pointed out that there is a case that the activation of astrocytes is induced to protect the neurons^34^. In addition, it is natural to consider that the decrease in the PVMs and the enhancement of ER stress markers are the results of clearance of waste products including denatured proteins to protect the brain tissue from their excessive accumulation. In fact, the ER stress promotes phagocytosis of macrophages to remove wastes and foreign matters^35^.

**Figure 3:**
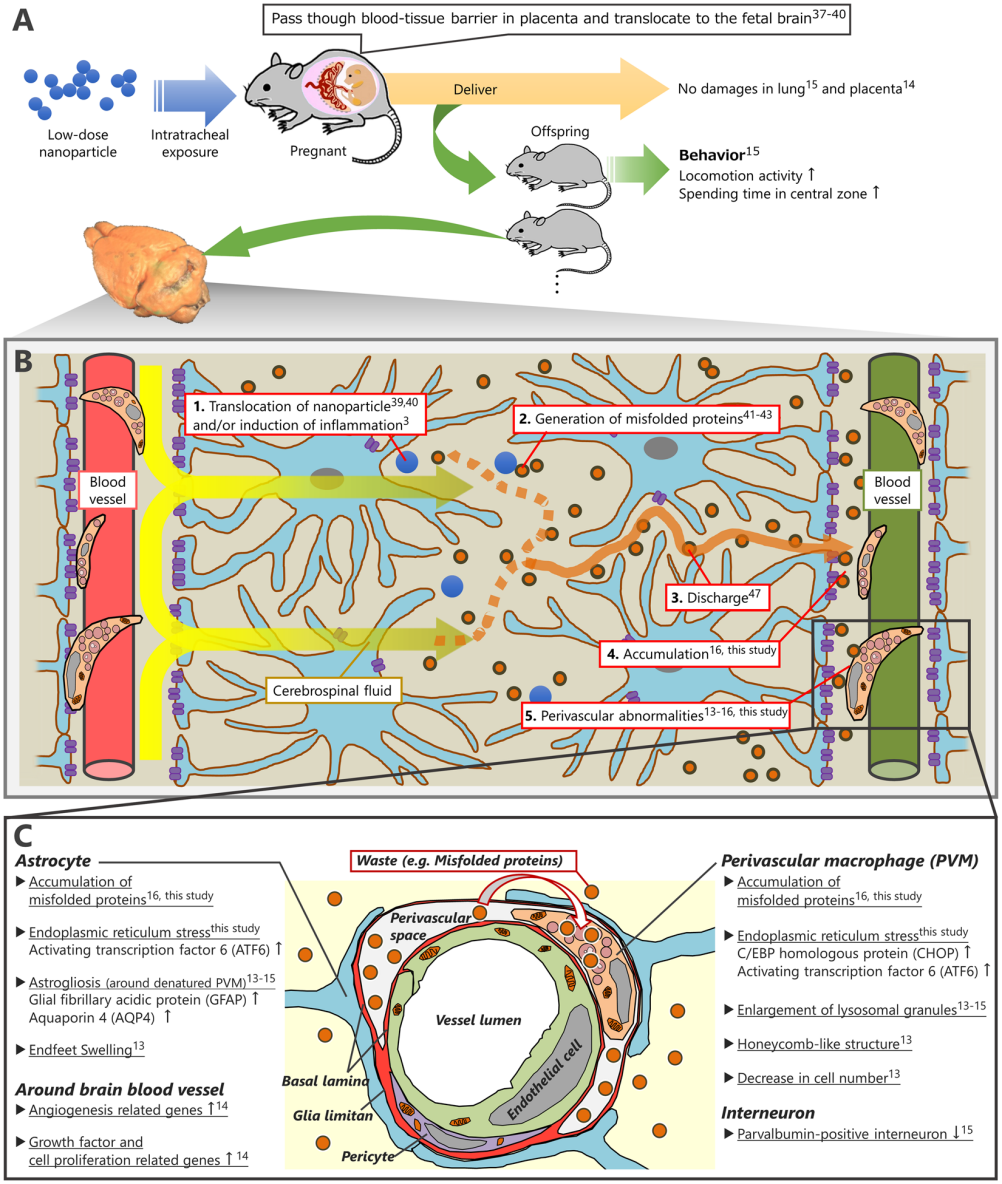
Potential mechanism of developmental neurotoxicity induced by nanoparticles based on the present and previous findings. (**A**) Nanoparticles invade into the pregnant mouse body through the respiratory tract and translocate to the foetal brain^37,40^, where they induce behavioural changes in the offspring even at low doses that do not adversely affect the maternal lungs^15^ and placenta^14^. (**B**) Nanoparticles translocated into the offspring brain^39,40^ react with proteins and thus change the protein secondary structures^42,43^. Neuroinflammation and oxidative stress following maternal exposure to nanoparticles may also cause protein misfolding^45^. Misfolded proteins are discharged by the cerebrospinal fluid and accumulate around brain blood vessels^16,47^. Accumulated misfolded proteins induce histopathological and functional damages in the brain blood vessels, glial cells, and neurons^13–16^. (**C**) Resulting histopathological and functional damages around brain blood vessels in the offspring induced by maternal exposure to low doses of nanoparticles^13–16^. (B) Background image is adapted from Reference No.^58^.

Previously, it has been shown that cellular uptake of nanoparticles does not directly induce ER stress^36^. Thus, it is worth discussing the relationship between localization of nanoparticles and induction of ER stress around brain blood vessels following maternal exposure to nanoparticles. Nanoparticles, owing to their small size, can translocate to the fetal brain through the blood placental barrier^37,38^ and immature blood brain barrier of the fetus^39,40^. Although nanoparticles do not deposit around the brain blood vessels but rather in brain parenchyma far from blood vessels^39,40^, brain perivascular tissues respond most sensitively to maternal exposure to nanoparticles^13,14,16^. Misfolded proteins may work as mediators between nanoparticles in the parenchyma and induction of ER stress in the perivascular tissues. Conformational changes in protein secondary structures frequently occur in response to physicochemical stimuli^41^. Surface reactions on nanoparticles also promote protein conformational changes by acting as a catalyst^42^ and, in particular, induce loss of α-helix and formation of β-sheet structures of intra- and extracellular proteins^43^. In fact, nanoparticles have been isolated from amyloid plaque cores from the brains of Alzheimer’s disease patients^8,44^. Further, protein misfolding is also led to neuroinflammation^45^, and maternal exposure to nanoparticles causes neuroinflamation^3^. The misfolded proteins on the nanoparticles may also propagate further protein misfolding, such as prion protein^46^. The evidence suggests that misfolded proteins are extremely likely to be generated and increased by nanoparticle exposure. Moreover, the central nervous system possesses a clearance system for misfolded proteins in the perivascular areas^47^. Misfolded proteins associated with neurodegenerative diseases are released and accumulate in the perivascular space, the most downstream of clearance route in the central nervous system, through the flow of cerebrospinal fluid^47^. In particular, PVMs and astrocytes have critical roles in the clearance of misfolded proteins. For example, PVMs phagocytose misfolded proteins including Aβ in the perivascular space^48,49^. Astrocytes congregate at sites of Aβ deposition and remove it from the brain in early pathogenesis of Alzheimer’s disease^50^. Thus, the observed increase in misfolded proteins in perivascular areas following maternal CB-NP exposure is likely to result from the clearance system. Based on these findings of the present and previous research, we can propose a potential principle of toxic mechanisms induced by maternal exposure to nanoparticles as follows (steps 1–5): Nanoparticles that enter pregnant mice invade the brain parenchyma of offspring far from blood vessels (1) and generate misfolded proteins via surface reactions and/or neuroinflammation (2). These misfolded proteins are discharged by cerebrospinal fluid (3) and accumulate in the perivascular space (4), subsequently inducing ER stress in PVMs and astrocytes around blood vessels (5). This ultimately leads to an induction of perivascular abnormalities such as astrogliosis (Fig. 3).

Some limitations merit discussion regarding this potential principle proposed by the current study. First, the increase in the β-sheet signal may be not only owing to the increase in protein misfolding but also by the protein secretion from the reactive astrocytes. The origin of the misfolded proteins has not been clarified yet and particularly needs to be investigated in future works to reveal the mechanisms. Also, the ER stress marker may be upregulated by misfolded proteins as well as high protein secretory load of the reactive astrocytes and PVMs. However, our proposed mechanism is supported by the observation of PVM denaturation which is sensitive to foreign substances and endogenous waste products including misfolded proteins^13^. Second, the translocated dose of CB-NP in the developing brains was not determined, because we have no technique to quantitatively analyze the distribution of nanoparticles composed of carbon in the biological tissues. Nanoparticles can pass through the placental barrier^37^ and the incomplete blood-brain barrier of fetal brains^39,40^, but only a small fraction do. Further investigations are needed to determine the translocated particle dose in the brain of offspring by using stable^51^ or radioactive isotopes^52^, fluorophores^53,54^, or ones composed of detectable elements^39^ following maternal exposure.

Since ER stress were observed in offspring at 6 weeks after birth, maternal CB-NP exposure may lead to a persistent decline in misfolded protein clearance capacity due to chronic perivascular abnormalities. Eventually, excessive accumulation of misfolded proteins is likely to result in neuronal injury. Previous studies have displayed that maternal CB-NP inhalation (5 min/day to 0, 4.6 or 37 mg/m^3^, gestation day 4–18) caused an injury of parvalbumin-positive interneurons, one of the most vulnerable neurons, and abnormal behavior including an increase in locomotor activity and a decrease in the fear cognition associated with a decrease in the interneurons^15^. The injury of the interneurons occurred in parallel with astrogliosis following maternal CB-NP inhalation^15^. Thus, neuronal injury may be induced by perivascular abnormalities and ER stress due to the accumulation of misfolded proteins. In fact, changes in the function of brain blood vessels are commonly observed prior to neurodegeneration, neuronal deposition of misfolded proteins, and onset of symptomatic neurodegenerative diseases^18,19^. Additionally, because ER stress in macrophages, especially CHOP upregulation, exacerbates cerebrovascular damage^55^, chronic ER stress in PVMs induced by maternal CB-NP exposure may promote long-term perivascular abnormalities and neuronal injury. Furthermore, the chronic ER stress and perivascular abnormalities were induced by low-dose exposure to CB-NP during pregnancy, while this dose not adversely affect placenta or maternal pulmonary organs^14,15^. The vulnerability of the developing brain to low-dose nanoparticle exposure during embryonic period may be exacerbated by the self-propagation and gradual spreading of toxic species including misfolded proteins in the brain, like a butterfly effect^56^. Therefore, nanoparticles in the brain may trigger the generation, propagation, and accumulation of misfolded protein around blood vessels, and the induction of chronic ER stress, thereby potentially resulting in perivascular abnormalities, neurodegeneration, and decline of clearance capacity.

## Conclusion

Overall, this is the first animal study demonstrating that maternal exposure to nanoparticles causes ER stress in cells of the central nervous system of the offspring. ER stress was particularly high in PVMs and reactive astrocytes around brain blood vessels, and was coincident with the accumulation of β-sheet-rich proteins regarded as misfolded proteins. At present, cytotoxicity associated with inflammation and oxidative stress has been considered to be the main mechanism underlying the developmental neurotoxicity of particulate air pollution^57^. Our results from the present study may support perivascular ER stress via protein misfolding as an additional mechanism to be considered. These mechanisms may help provide potential insight into the epidemiological observation that particulate air pollution disturbs brain development, induces abnormal cognitive functions, and predisposes to neurodegenerative diseases^6,7,11^. We propose that inhibition of maternal exposure to particulate air pollution may prevent the accumulation of misfolded proteins, induction of ER stress with perivascular abnormalities and neurodegeneration, and development of brain disorders. Also, further studies into the Adverse Outcome Pathways associated with the mechanisms may lead to the discovery of new therapeutic targets for the treatment of abnormal brain development and related disorders. In particular, results of the present study suggest that proteins whose conformation is easily changed by nanoparticles, β-sheet rich proteins that accumulate around brain blood vessels, and molecules that control waste clearance in the brain are strong candidates for target molecules. Identification of these biomolecules may help prevent neurodegenerative diseases caused by particulate air pollution.

## Data Availability

All data necessary to evaluate the conclusions in the paper are available in the paper. The datasets used and analysed during the current study are available from the corresponding author on reasonable request.
